# Prevalence of Porcine Circoviruses in Slaughterhouses in Central Shanxi Province, China

**DOI:** 10.3389/fvets.2022.820914

**Published:** 2022-05-23

**Authors:** Weidong Yue, Yilei Li, Xinrong Zhang, Junping He, Haili Ma

**Affiliations:** College of Veterinary Medicine, Shanxi Agricultural University, Jinzhong, China

**Keywords:** porcine circovirus type 3, porcine circovirus type 4, prevalence, co-positive, PCR

## Abstract

**Background:**

Porcine circovirus disease is currently the greatest threat to pig farming. Four main porcine circovirus genotypes are circulating worldwide.

**Objective:**

The study aimed to assess the prevalence of porcine circovirus genotypes in the central part of Shanxi province.

**Methods:**

We investigated the prevalence of porcine circovirus type 2 (PCV2), porcine circovirus type 3 (PCV3), and porcine circovirus type 4 (PCV4). Porcine circoviruses were analyzed by polymerase chain reaction (PCR) in the lung tissues of 180 pigs from 7 slaughterhouses in central Shanxi, China.

**Results:**

The prevalence of PCV2, PCV3, and PCV4 were 56.8, 80, and 9.4%, respectively, and the negative rate was 10% for all three pathogens. The co-infection with PCV2 + PCV3, PCV2 + PCV4, PCV3 + PCV4, and PCV2 + PCV3 + PCV4 were 47.2, 7.4, 7.4, and 5.6%, respectively. Among PCV4-positive samples, the positive rate of PCV4 + PCV2 was 52.9% (9/17), whereas that of PCV4 + PCV3 was 100% (17/17). On the other hand, PCV2 and PCV3 were detected in 57.1% (93/163) and in 78.5% (128/163) of PCV4-negative samples, respectively. Phylogenetic analysis demonstrated that PCV2, PCV3, and PCV4 were not in the same clade and were distant from each other.

**Conclusion:**

The high positive rates of PCV3, PCV2 + PCV3, and PCV3 + PCV4 suggest that PCV3 may play a decisive role in PCV2 and PCV4 infections. Therefore, further control of PCV3 is needed to reduce the spread of the virus.

## Introduction

Circovirus is a small non-enveloped virus with a circular icosahedral single-stranded DNA genome and can cause infections in different animals (1–3). Porcine circovirus type 1 (PCV1) and porcine circovirus type 2 (PCV2) were the earliest circoviruses found in pigs and have been prevalent for more than 20 years (4, 5). PCV1 is considered nonpathogenic to pigs (6). On the other hand, PCV2 can cause post-weaning multi-systemic easting disease syndrome (PMWS), respiratory diseases, porcine dermatitis, and nephropathy syndrome (PDNS) and has spread throughout pig farms worldwide (7, 8). Porcine circovirus type 3 (PCV3) and porcine circovirus type 4 (PCV4) are newly identified porcine circoviruses. PCV3 was first reported in PDNS pigs in the United States in 2016. Moreover, PCV3 has been detected in pigs with respiratory diseases and multi-system inflammation (9–11). Porcine circovirus type 4 (PCV4) was first detected in Hunan, China, in 2019 (12). The first case of PCV3 was reported in China in 2017; since then, PCV3 has been reported in Jiangxi, Fujian, Shandong, and Liaoning provinces, which are major pig-producing provinces in China (13–16). Furthermore, PCV3 has been reported in several countries, including China, the United States, Italy, Korea, Spain, and Russia (9, 17–22). At present, PCV4 has been reported in Hunan, Henan, Guangxi, and Shanxi provinces in China (12, 23, 24).

In a previous study, the positive rate of PCV3 was 44.2% in the United States (10). In studies in Europe, the positive rate of PCV3 was 75% in Germany and 65% in Poland (14, 18). On the other hand, a positive rate of 44.2% has been reported in Korea (20). In China, high PCV3 positive rates have been reported in Anhui province (20%), Jiangsu province (26.7%), Zhejiang province (23.3%), Chongqing City (16.7%), and Hunan province (45.9%) (16, 25, 26). PCV4 was detected for the first time in Hunan province with a positive rate of 12.8%. In a retrospective study by Tian et al. in Henan and Shanxi provinces from 2018 to 2019, the positive rate of PCV4 was 25.4% (23). A retrospective analysis by Sun et al. in Guangxi from 2015 to 2019 found that the positive rate of PCV4 was 9.1%, and the co-infection with PCV2, PCV3, and PCV4 was 69.2% (24).

In this study, we investigated the prevalence of PCV2, PCV3, and PCV4 in central Shanxi. The rates of co-infection with PCV3 + PCV2, PCV3 + PCV4, PCV2 + PCV4, and PCV2 + PCV3 + PCV4 were also examined.

## Materials and Methods

### Sample Collection

**Figure 1:**
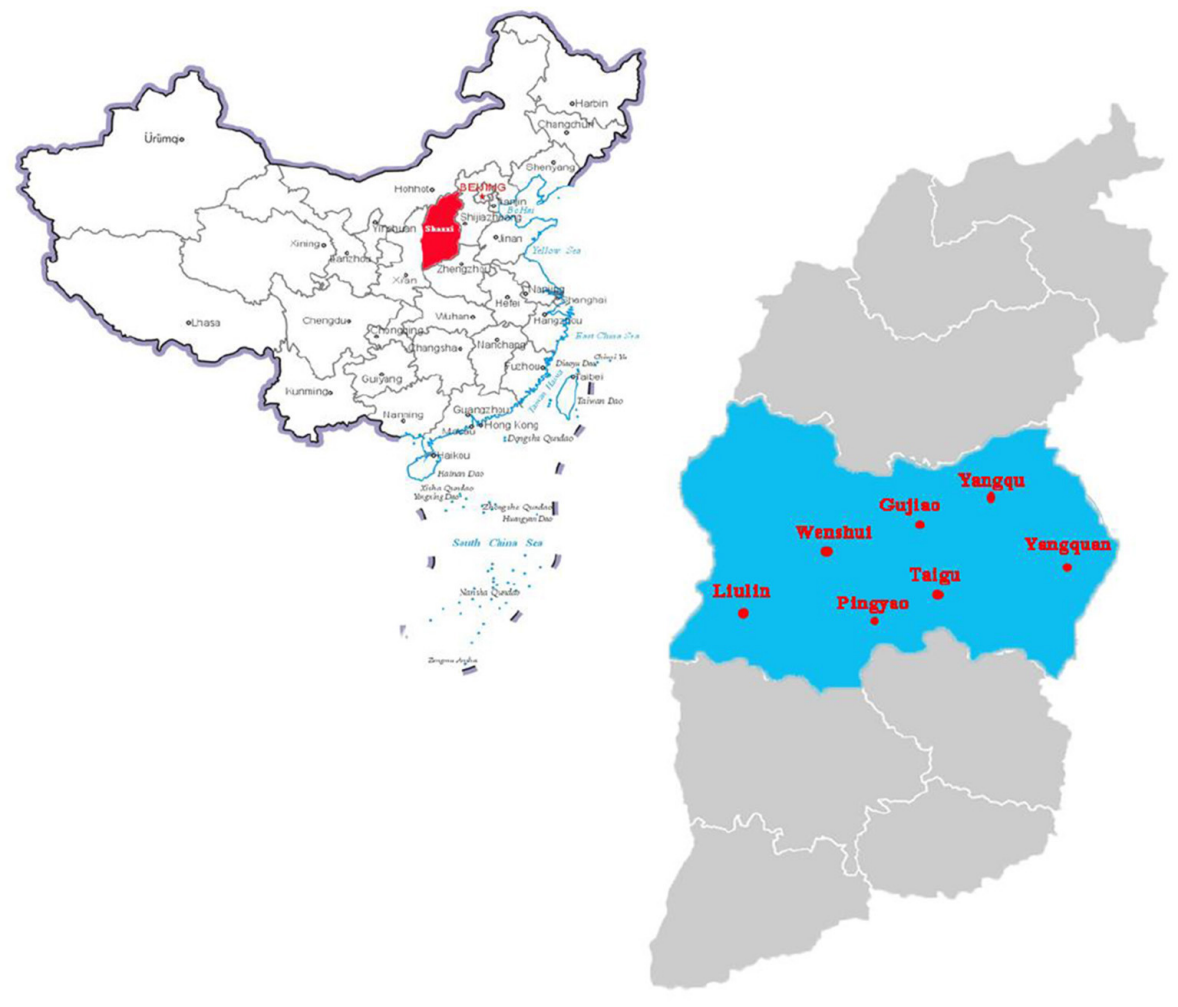
Pig sampling locations in center of Shanxi Province, China. The standard map of China is obtained from the national standard map system (http://bzdt.ch.mnr.gov.cn/). Red area indicates where Shanxi province is in China. The blue area indicates the sampling collection cities, the scarlet letter indicates where the samples were collected in Shanxi province, China.

Lung tissue samples (*n* = 180) were collected from 7 pig slaughterhouses, accounting for 17.5% of all slaughterhouses (40 slaughterhouses) in the central region of Shanxi province, China, in 2019–2020 (Figure 1). The daily slaughter capacity of each slaughterhouse was 1,000–2,000 animals. The tissue samples were randomly collected from slaughtering lines, placed in sterile polyethylene (PE) bags containing tissue protection solution, and transported back to the laboratory in a cooled storage incubator (4–10°C) for sterile separation. The tissue was washed with sterile saline, homogenized, and subjected to DNA extraction.

### DNA Extraction and Polymerase Chain Reaction

Tissue samples were extracted using the TIANamp Genomic DNA Kit (Tianen, Beijing, China). Three additional pairs of PCR primers were used, as shown in Table 1. The PCR reaction volume contained 1.5 μL of DNA, 1 μL of primer pairs (10 μM), 10 μL of 2 × Taq Plus Master Mix II (Vazyme, Nanjing, China), and 6.5 μL of ddH_2_O. PCR cycle conditions for PCV2 were as follows: pre-denaturation at 94°C for 4 min, followed by 30 cycles at 94°C for 1 min, 55°C for 1 min, and 72°C for 1 min, and a final extension step at 72°C for 7 min. PCR cycle conditions for PCV3 were as follows: pre-denaturation at 94°C for 5 min, followed by 34 cycles at 94°C for 30 s, 55°C for 30 s, and 72°C for 30 s, and a final extension step at 72°C for 7 min. PCR cycle conditions for PCV4 were as follows: pre-denaturation at 95°C for 4 min, followed by 35 cycles at 95°C for 20 s, 58°C for 20 s, and 72°C for 25 s, and a final extension step at 72°C for 10 min (14, 23).

**Table 1:** Primer sequences used porcine circovirus diseases.

**Primer name**	**Primer sequence**	**Reference**
PCV2	F:5′-TTTAGG GTTTAAGTGGGG GGTC-3′	(27)
	R:5′-CCGGATCCATGACGTACCCAAGGAGGCG-3′	
PCV3	F:5′-TGCTACGAGTGTCCTGAAGAT-3′	(14)
	R:5′-CTTCTCCGCAACTTCAGTCAG-3′	
PCV4	F:5′-GTTTTTCCCTTCCCCCACATAG-3′	(23)
	R:5′-ACAGATGCCAATCAGATCTAGGTAC-3′	

*PCR, polymerase chain reaction; PCV2, Porcine circovirus type 2; PCV3, Porcine circovirus type 3; PCV4, Porcine circovirus type 4*.

To identify swine pathogens such as *Mycoplasma hyopneumoniae* (M.hyo), porcine reproductive and respiratory syndrome virus (PRRSV), *Haemophilus parasuis* (Hps), pseudorabies virus (PRV), and swine influenza virus (SIV) in the tissue samples, reverse transcription, and PCR assays were performed as previously described (17).

### Phylogenetic Analysis of PCV2, PCV3, and PCV4 Strains

Data on the Cap gene (*n* = 14) of PCV2, PCV3, and PCV4 strains isolated from different geographical locations were retrieved from reference sequences in the NCBI database. Three strains of PCV2, three strains of PCV3, and two strains of PCV4 were included in this study. Multiple sequence alignments of PCV2, PCV3, and PCV4 strains, including the novel PCV2, PCV3, and PCV4 strains identified in this study, were performed using the Clustal W method (DNA-STAR Inc., Madison, WI, USA). Phylogenetic relationships were assessed using the neighbor-joining method, the p-distance model with 1,000 bootstrap replicates, and the remaining default parameters with MEGA software (MEGA 6.06) (28).

### Data Analysis

The PCR results were analyzed as qualitative (positive/negative) data. All PCR products were analyzed by 2% agarose gel electrophoresis and stained with GelRed™ (Biosharp, Hefei, China). The bands were visualized under ultraviolet illumination. The SPSS software independence test was used to analyze the correlation between the PCV4 with PCV2 and PCV3. GraphPad Prism 5.0 (GraphPad, San Diego, CA, USA) was used for plotting.

## Results and Discussion

Samples positive for Hps, M.hyo, PRRSV, and SIV were found; however, no sample was positive for PRV. The prevalence of PCV2, PCV3, and PCV4 were 56.7, 80, and 9.4%, respectively, in this study (Table 2). According to this study's findings, the prevalence of PCV2 and PCV3 was over 50%, and the positive sample of PCV3 was up to 80% in central Shanxi. Similarly, we found a high prevalence of PCV3 and PCV2 in the central region of Shanxi. Due to huge economic losses caused by porcine circovirus-associated disease (PCVAD), porcine circoviruses have attracted attention in the swine industry. PCV1 is considered non-pathogenic to pigs, and PCV2 may be controlled through PCV2 vaccination in pigs (29). Many countries, including China, South Korea, Thailand, and Poland, have reported similar findings. Recent studies reported that PCV3 could cause not only diseases in the respiratory system, reproductive system, and nervous system, but also diarrhea in pigs (30). Therefore, the high prevalence of PCV3 in central Shanxi could endanger the health of pigs in the area. PCV4 was first reported in Hunan, China in 2019 (12), and was subsequently detected in several cities in China (23, 24). In Italy and Spain, PCV4 failed to be detected in 2020 (31). PCV4 was detected at a rate of 3.28% in six provinces in Korea in 2019–2020 (32). The presence of PCV4 in different geographic locations implied the active circulation of the virus. Taken together, these studies demonstrated the prevalence of PCV4 in pigs. Although PCV4 was first reported at the end of 2019, PCV4 was detected in clinical samples dating back to the beginning of 2019 in our study. In other retrospective studies, PCV4 has been detected in samples as early as 2016. These are the earliest PCV4-positive samples identified, thus, far (33). Nevertheless, whether PCV4 is a newly emerging PCV or one that has infected pigs for some time requires further investigation.

**Table 2:** The positivity rate of PCV2, PCV3, and PCV4 in central Shanxi Province.

**City**	**Positivity rate (** ***n*** **) %**		
	**PCV2**	**PCV3**	**PCV4**
Wenshui	(16/28) 57	(28/28) 100	(11/28) 0
Liulin	(16/26) 61	(22/26) 83	(0/26) 0
Pingyao	(16/26) 61	(26/26) 100	(3/26) 11.54
Taigu	(18/23) 78	(16/23) 69	(0/23) 0
Yangquan	(6/28) 21	(20/28) 71	(1/28) 3.57
Gujiao	(21/27) 77	(18/27) 66	(2/27) 7
Yangqu	(9/22) 40	(14/22) 63	(0/22) 0
Total	(102/180) 56.7	(144/180) 80.0	(17/180) 9.4

*PCV2, Porcine circovirus type 2; PCV3, Porcine circovirus type 3; PCV4, Porcine circovirus type 4*.

**Figure 2:**
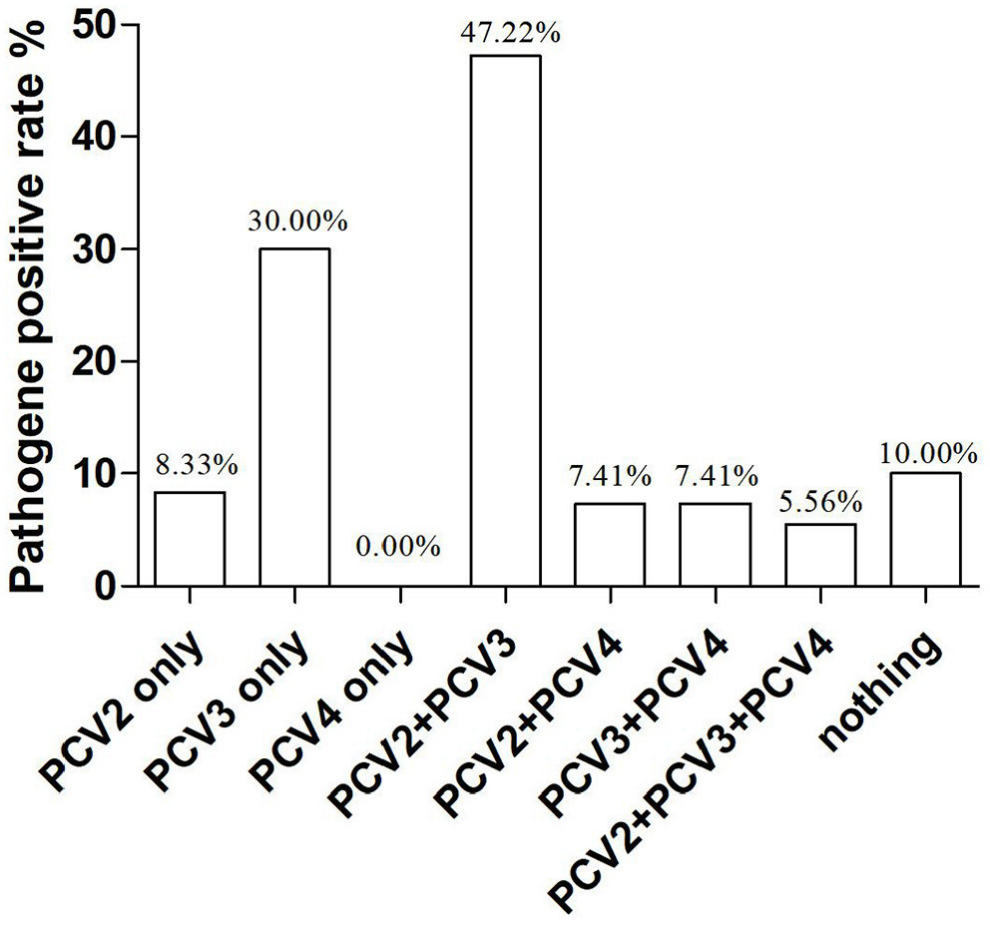
Proportions of major pathogens in PCR-positive porcine samples.

In our study, co-infections with PCV2, PCV3, and PCV4 were frequently detected in the lung tissue of piglets. To investigate the correlation between PCV4 infection and PCV2 and PCV3 infections in pigs, we determined the co-infection with PCV4 and PCV2 and/or PCV3. The rates of co-infection with PCV2 + PCV3, PCV2 + PCV4, PCV3 + PCV4, and PCV2 + PCV3 + PCV4 were 47.2, 7.41, 7.4, and 5.6%, respectively (Table 3, Figure 2). The negative rate was 10% for all three pathogens (Figure 2). The individual positive rates of PCV2, PCV3, and PCV4 were 8.3, 30, and 0.0%, respectively (Figure 2). In general, instances of co-infection are common in pigs, and they can exacerbate disease severity (34). Among the swine pathogens, co-infection with PCV2 + PCV3 has been the most commonly reported in pig farms. Some studies showed that the co-infection rates of PCV2 + PCV3 were 6.8% from 2015 to 2018 and 19.7% from 2018 to 2020 in China (35, 36). Moreover, Wang et al. evaluated cases of co-infection in the Midwest of the United States from 2016 to 2018 and found that the rate of co-infection with PCV2 + PCV3 gradually increased from 3.4 to 16.1% (29). Notably, the positive rate of PCV2 + PCV3 in the sera from clinically healthy fattening pigs in nine European countries was 3% (37). In addition, a report from South Korea revealed that the positive rate of PCV2 + PCV3 was 28.3% (38). The results suggest that co-infection with PCV2 and PCV3 is widespread, showing a gradual upward trend in recent years.

**Table 3:** Co-positivity rate of PCV2, PCV3, and PCV4 in central Shanxi Province.

**City**	**Pathogen positivity rate (** ***n*** **) %**			
	**PCV2 + PCV3**	**PCV2 + PCV4**	**PCV3 + PCV4**	**PCV2 + PCV3 + PCV4**
Wenshui	(16/28) 57	(28/28) 100	(11/28) 0	(5/28) 17
Liulin	(16/26) 61	(22/26) 83	(0/26) 0	(2/26) 7
Pingyao	(16/26) 61	(26/26) 100	(3/26) 11	(0/26) 0
Taigu	(18/23) 78	(16/23) 69	(0/23) 0	(0/23) 0
Yangquan	(6/28) 21	(20/28) 71	(1/28) 3	(1/28) 3
Gujiao	(21/27) 77	(18/27) 66	(2/27) 7	(2/27) 7
Yangqu	(9/22) 40	(14/22) 63	(0/22) 0	(0/22) 0
Total	(102/180) 47.2	(144/180) 7.4	(17/180) 7.4	(10/180) 5.6

*PCV2, Porcine circovirus type 2; PCV3, Porcine circovirus type 3; PCV4, Porcine circovirus type 4*.

**Figure 3:**
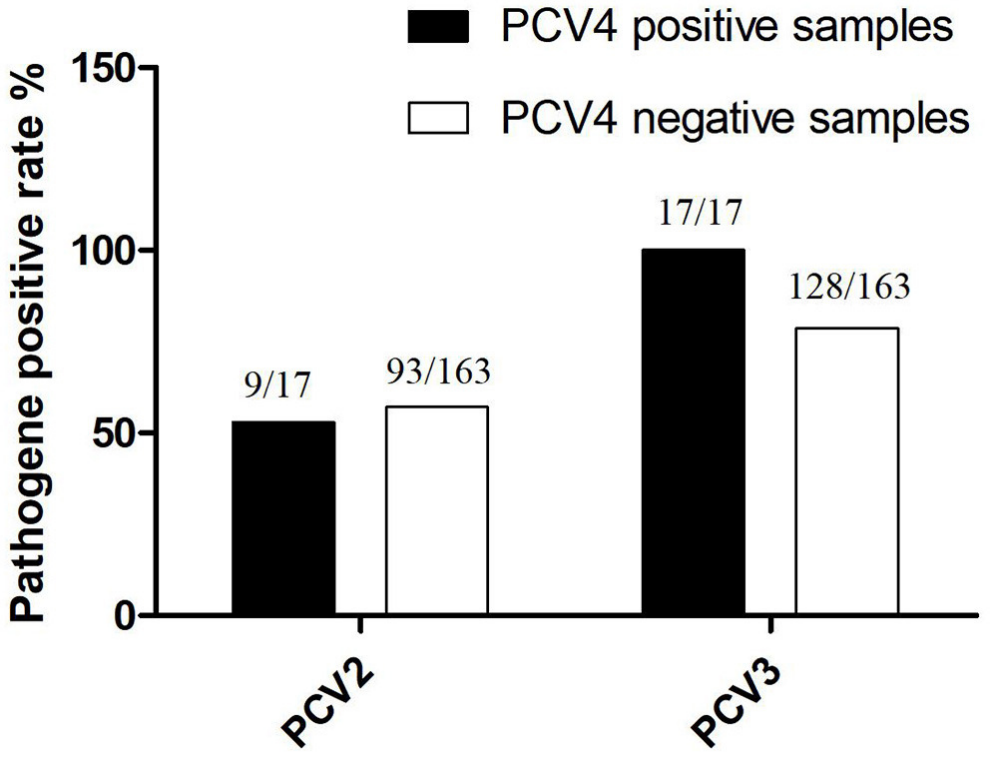
The co-positive rate of PCV4 with PCV2 and PCV3.

Among PCV4-positive samples, the positive rate of PCV4 + PCV2 was 52.94% (9/17), whereas that of PCV4 + PCV3 was 100% (17/17). On the other hand, PCV2 and PCV3 were detected in 57.1% (93/163) and 78.5% (128/163) of PCV4-negative samples, respectively (Figure 3). The results confirmed significant correlations between PCV3 and PCV4 (*P* = 0.001, *P*< *0.0*5), PCV2, and PCV3 (*P* = 0.01, *P* < 0.05). The findings indicated a possible correlation between PCV4 and PCV3 infections; however, further investigation is needed. In this study, a high positive rate of PCV3 was observed in the sample size without specific geographical patterns. Moreover, the rate of co-infection with PCV3 + PCV4 was higher than that with PCV2 + PCV4. The results suggest that PCV3 may play a decisive role in PCV2 and PCV4 infections.

Considering the findings, it is necessary to analyze the genetic relationships among PCV2, PCV3, and PCV4. Because the isolation and culture of PCV3 and PCV4 are difficult, most recent studies have focused on the genetic characteristics of the virus. The ORF2 gene encodes the capsid (Cap) protein of porcine circoviruses. The Cap protein, a major structural protein, determines the antigenic characteristics of circoviruses (14). Therefore, positive samples were further sequenced, followed by phylogenetic analysis of PCV2, PCV3, and PCV4 ORF2 genes.

**Figure 4:**
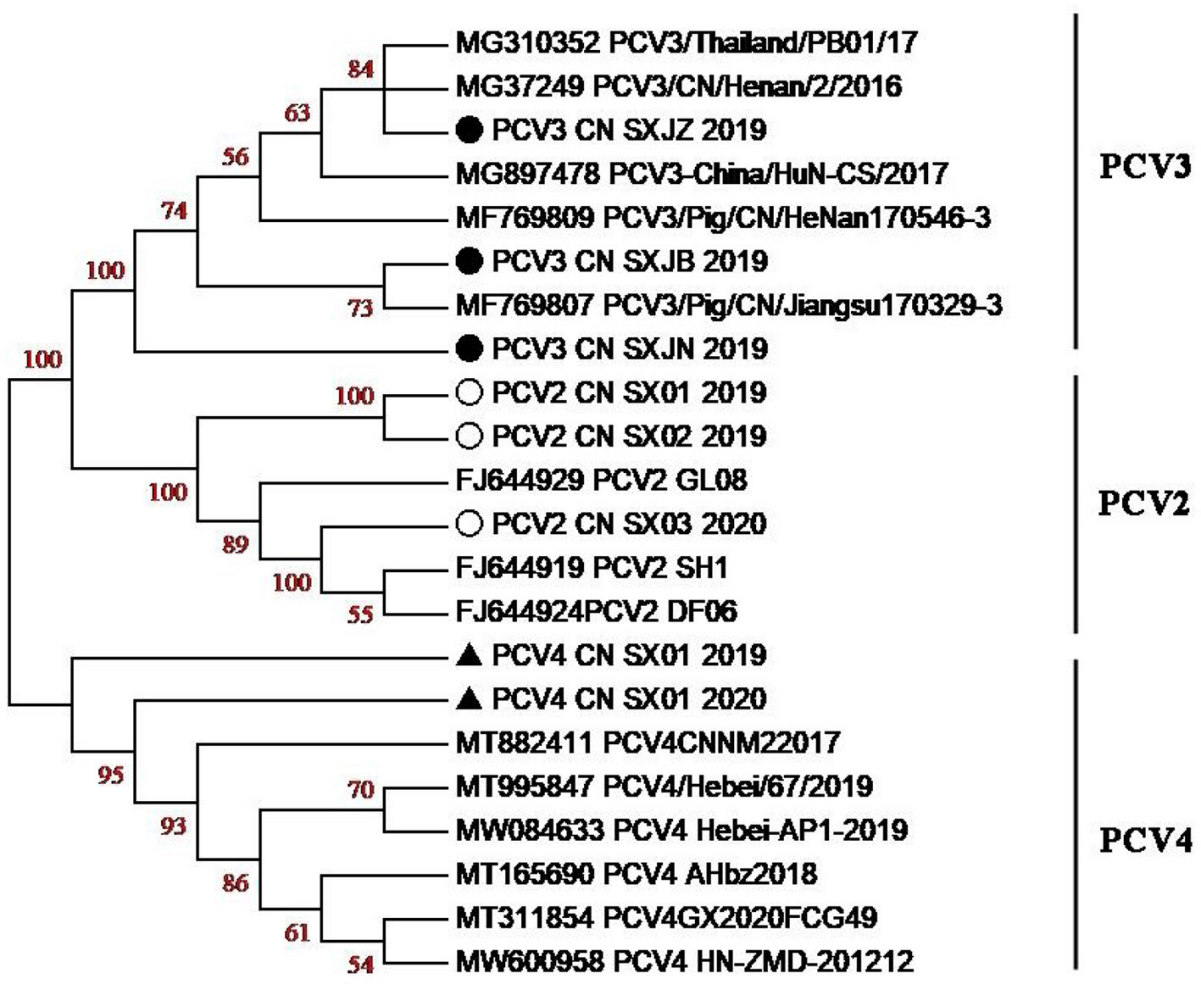
Phylogenetic tree based on the ORF2-encoding gene of PCV2, PCV3, and PCV4 strains. The PCV strains clustered into three subgroups, namely, PCV2, PCV3, and PCV4. Black solid circles (•) indicate PCV3 strains, hollow circle (°) indicates PCV2 strains, black solid triangle (▴) indicates PCV4 strains in this study, respectively.

Phylogenetic tree analysis based on the ORF2 gene showed that PCV3 CN SXJB 2019 was closely related to PCV3/pig/CN/jiangshu170329-3 (MF769807). In addition, PCV2 CN SX02 2020 was closely related to PCV2 SH1 (FJ644919), and PCV4 CN SX01 2020 was closely related to PCV4 CNNM2017 (MT882411). The results demonstrated that PCV4 and PCV3 were not in the same clade and were distant from each other (Figure 4). However, the limited number of sequences did not allow an independent evolutionary analysis of PCV4. Furthermore, previous studies confirmed that PCV2 and PCV3 could be transmitted to non-porcine hosts, possibly through cross-species transmission routes (39). The likelihood of this occurring with PCV4 needs to be further investigated.

## Conclusion

We investigated the prevalence of PCV2, PCV3, and PCV4 in central Shanxi in 2019–2020. The results suggest that PCV3 may play a decisive role in PCV2 and PCV4 infections. Therefore, further control of PCV3 is needed to reduce the spread of the virus.

## Data Availability Statement

The original contributions presented in the study are included in the article/supplementary material, further inquiries can be directed to the corresponding authors.

## Ethics Statement

This study was conducted in accordance with the ethical guidelines of Shanxi Agricultural University (SAU; Taigu, China). Prior to the commencement of the study, ethical approval was granted by the Institutional Animal Care and Use Committee, Shanxi Agricultural University (Approval No. SXAU-EAW-2019P002017).

## Author Contributions

JH and HM: conceptualization. YL: methodology, investigation, data curation, and avisualization. XZ: software. HM, JH, and WY: validation. WY: formal analysis, writing—original draft preparation, and writing—review and editing. HM: resources, supervision, and funding acquisition. JH: project administration. All authors have read and agreed to the published version of the manuscript.

## Funding

This research was supported by funding from the Shanxi Agricultural University Academic Restoration project (Grant No. 2020xshf15); the Key Research and Development Project Key Program of Shanxi Province, China (Grant No. 201703D211001).

## Conflict of Interest

The authors declare that the research was conducted in the absence of any commercial or financial relationships that could be construed as a potential conflict of interest.

## Publisher's Note

All claims expressed in this article are solely those of the authors and do not necessarily represent those of their affiliated organizations, or those of the publisher, the editors and the reviewers. Any product that may be evaluated in this article, or claim that may be made by its manufacturer, is not guaranteed or endorsed by the publisher.

## Abbreviations

PCV-1: porcine circovirus type 1
PCV-2: porcine circovirus type 1
PCV-3: porcine circovirus type 3
PCV-4: porcine circovirus type 4
PCR: polymerase chain reaction
NT: nucleotides.
